# Transitional impact of short‐ and long‐term outcomes of a randomized controlled trial to evaluate laparoscopic versus open surgery for colorectal cancer from Japan Clinical Oncology Group Study JCOG0404

**DOI:** 10.1002/ags3.12245

**Published:** 2019-03-26

**Authors:** Shoichi Fujii, Tomonori Akagi, Masafumi Inomata, Hiroshi Katayama, Junki Mizusawa, Mitsuyoshi Ota, Shuji Saito, Yusuke Kinugasa, Shigeki Yamaguchi, Takeo Sato, Seigo Kitano, The Japan Clinical Oncology Group

**Affiliations:** ^1^ Department of Gastroenterological Surgery Yokohama City University Medical Center Yokohama Japan; ^2^ Department of Gastroenterological and Pediatric Surgery Oita University Oita Japan; ^3^ Japan Clinical Oncology Group Data Center/Operations Office National Cancer Center Tokyo Japan; ^4^ Division of Colon and Rectal Surgery Shizuoka Cancer Center Shizuoka Japan; ^5^ Department of Surgery Kitasato University Hospital Sagamihara Japan

**Keywords:** colorectal cancer, laparoscopic colectomy, randomized controlled trial, transitional impact

## Abstract

**Background:**

The JCOG0404 randomized controlled trial conducted to compare laparoscopic surgery (LAP) with open surgery (OP) for stage II/III colon cancer showed better short‐term outcomes and equal long‐term outcomes of LAP versus OP. Technical instrumentation of surgery and anticancer agents given during the registration period might have affected the outcomes.

**Aim:**

To evaluate outcomes according to the registration periods.

**Methods:**

The overall registration period was divided into three periods (first: 2004‐2005, second: 2006‐2007 and third: 2008‐2009). Short‐term and long‐term outcomes were compared between registration periods.

**Results:**

In total, 1057 patients were registered. Numbers of patients undergoing each approach for each of the three periods (1st/2nd/3rd) were 528 for OP (106/244/178) and 529 for LAP (106/246/177). Operation time (minutes) did not change between the periods for OP (160/156/161) or LAP (205/211/219). Blood loss (mL) gradually decreased in the latter two periods: (119/80/75) for OP and (35/28/25) for LAP. Incidence of complications (%) decreased in the latter periods for OP (27.6/20.3/21.3), whereas that for LAP remained consistently low (14.3/14.8/13.6). There was no particular trend in 5‐year overall survival and recurrence‐free survival depending on the period regardless of treatment. D3 dissection rates were 95% or more for all periods in both groups.

**Conclusions:**

Operation time and survival rates did not change over time, whereas blood loss in OP improved in the latter periods. Quality control applied in this trial might have been effective in producing such safe endpoints. (ClinicalTrials.gov, number NCT00147134, UMIN Clinical Trials Registry, number C000000105.)

## 1 INTRODUCTION

At the beginning of this century, several randomized controlled trials for colorectal cancer from all over the world showed excellent short‐term results and equal long‐term results of laparoscopic surgery compared with open surgery.^1^, ^2^, ^3^, ^4^, ^5^, ^6^, ^7^, ^8^ Most of these trials showed that laparoscopic surgery for colon cancer has been accepted as a standard therapy. In Japan, the Japan Clinical Oncology Group (JCOG) conducted a randomized controlled trial to confirm the efficacy of laparoscopic surgery for stage II or III colon cancer (JCOG0404).^9^, ^10^, ^11^ The result of JCOG0404 was interpreted to be that laparoscopic surgery could be an acceptable treatment option for stage II or III colon cancer.

In terms of the quality control of this trial, our clinical question was whether the duration of this study could affect the clinical outcomes. The registration period in JCOG0404 ran from October 2004 to March 2009. According to the nationwide survey of laparoscopic surgery carried out by the Japan Society for Endoscopic Surgery (JSES), the spread of laparoscopic surgery for colorectal cancer increased approximately threefold during this registration period (from 4385 patients in 2004 to 14 032 patients in 2009).^12^ An operative procedure and new surgical devices such as ultrasonic apparatus and a bipolar sealing system for coagulation and incision were incorporated on a nationwide scale and spread during that time. The registration period of this study matched this spread of laparoscopic surgery. Additionally, improvements made to anticancer agents for patients with recurrent colorectal cancer during the recent 10 years might also influence survival after the occurrence of relapse.

Differences in the treatment results between the phases of a registration period is one of the most important factors in assessing the quality of a therapeutic technique in a clinical study. Registration periods of approximately 4.7 to 7 years have been required in past representative randomized controlled studies.^1^, ^2^, ^3^ However, evaluation of the results based on different periods within the overall registration period has not been reported in past randomized controlled studies.

Therefore, the purpose of the present study was to evaluate trial outcomes based on different periods within the overall registration period for JCOG0404. We analyzed the changes in short‐term and long‐term outcomes over time for each registration period and investigated whether quality of the surgical technique was maintained in this trial.

## 2 METHODS

### 2.1 Summary of JCOG0404

JCOG0404 was a multi‐institutional trial to confirm the non‐inferiority of laparoscopic surgery compared to open surgery for clinical stage II or III colorectal cancer. Eligibility criteria were as follows: histologically proven colon carcinoma; tumor located in the cecum, ascending, sigmoid, and rectosigmoid colon; clinical T3‐4a, N0‐2, M0; no multiple cancers; no double cancer; no bowel obstruction; tumor size of 8 cm or smaller; no history of chemotherapy and radiotherapy; no history of intestinal resection excluding appendectomy; age 20‐75 years old; and provision of written informed consent. Experience of 30 or more laparoscopic surgeries was indispensable as a participation regulation at the beginning of this study. The leading hospitals of laparoscopic colorectal cancer surgeries of JSES were selected by the principal investigator of study at the beginning of this study. After 2008, an endoscopically surgically qualified surgeon according to JSES was added as an indispensable board member. Submission of photographs of the resected field after D3 lymph node dissection, the specimen, and the skin incision in all patients was required for central review of the surgical procedure.^13^ Primary endpoint was overall survival. Secondary endpoints were relapse‐free survival, short‐term clinical outcomes, incidence of adverse events, and proportion of conversion from laparoscopic surgery to open surgery.^11^ This trial is registered with ClinicalTrials.gov, number NCT00147134, and UMIN Clinical Trials Registry, number C000000105.

### 2.2 Measured outcomes

Adequateness of D3 dissection was evaluated by central review. Cases with distant metastases or severe invasion to adjacent organs were excluded from the photographic analysis. Short‐term outcomes of operative time, blood loss, length of postoperative hospital stay, and incidence of early complications were analyzed. Early complications were defined as occurrences within 30 days of surgery. Terminology and grading of complications were described according to the Common Terminology Criteria for Adverse Events 3.0.^14^ Incidence of early complications included all grades in this study. Long‐term outcomes of overall survival and relapse‐free survival were compared between three periods within the overall registration period for each approach. Definitions of these outcomes are reported elsewhere.^11^

### 2.3 Statistical analysis

All patients were divided into one of three periods to assess the transitional impact of the trial. The first period was from October 2004 to December 2005; the second period was from January 2006 to December 2007; and the third period was from January 2008 to March 2009. Changes in continuous variables were analyzed by the Kruskal‐Wallis test, and categorical variables were analyzed by Fisher's exact test. The Kaplan‐Meier method was used to estimate overall survival and relapse‐free survival, and the log‐rank test was used to compare the three periods. A Cox proportional hazard model was used to estimate the hazard ratio (HR) of the latter two periods to the first period in overall survival and relapse‐free survival.

Overall survival and relapse‐free survival were analyzed by intention‐to‐treat. Short‐term outcomes of operative time and incidence of early complications were analyzed in the patients who had surgery as assigned, and those patients who did not undergo the assigned approach were excluded. All *P* values were two‐sided. All statistical analyses were carried out with SAS version 9.2.

## 3 RESULTS

### 3.1 Clinical characteristics

In total, 1057 patients were enrolled from October 2004 to March 2009 in JCOG0404. After randomization, eight patients in the open surgery group and four patients in the laparoscopic surgery group did not undergo their assigned surgery. Therefore, 1045 patients were analyzed for short‐term outcomes after excluding the 12 patients who underwent the alternative surgery.

**Figure 1:**
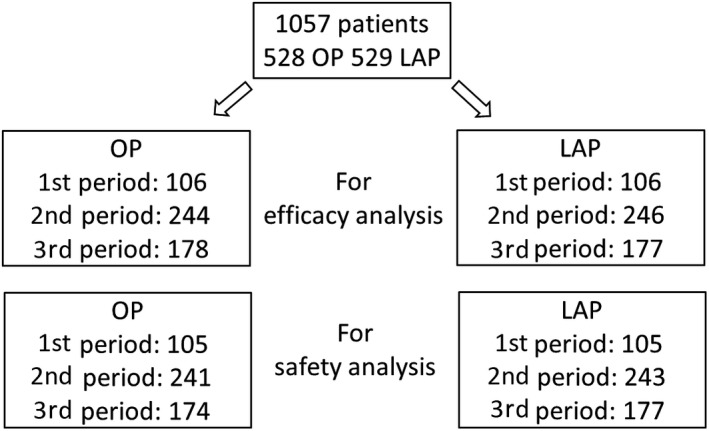
Study profile. First period: October 2004 to December 2005, second period: January 2006 to December 2007, third period: January 2008 to March 2009. LAP, laparoscopic surgery; OP, open surgery

For the analysis of efficacy, numbers of patients for each of the three periods for each approach were 528 for open surgery (1st period: 106, 2nd period: 244, 3rd period: 178) and 529 for laparoscopic surgery (1st period: 106, 2nd period: 246, 3rd period: 177). For the safety analysis, there were 520 patients for open surgery (1st period: 105, 2nd period: 241, 3rd period: 174) and 525 patients for laparoscopic surgery (1st period: 105, 2nd period: 243, 3rd period: 177) (Figure 1). There were no significant changes in relation to age, gender, body mass index (BMI), tumor location and clinical stage over time in both groups (Table 1).

**Table 1:** Clinical characteristics of patients in the present study

	OP			LAP		
	1st period	2nd period	3rd period	1st period	2nd period	3rd period
N	106	244	178	106	246	177
Age (median, y)	65	64	64	63	64	65
Gender (M:F)	63:43	143:101	106:72	54:52	134:112	94:83
BMI (median, kg/m^2^)	22.7	22.6	22.8	23.1	22.5	22.9
Tumor location (%)						
Cecum	11 (10.4)	26 (10.7)	19 (10.7)	9 (8.5)	24 (9.8)	13 (7.3)
Ascending colon	21 (19.8)	41 (16.8)	37 (20.8)	24 (22.6)	43 (17.5)	42 (23.7)
Sigmoid colon	45 (42.5)	118 (48.4)	72 (40.4)	51 (48.1)	123 (50.0)	76 (42.9)
Rectosigmoid colon	29 (27.4)	59 (24.2)	50 (28.1)	22 (20.8)	56 (22.8)	46 (26.0)
cStage (%)						
II	75 (71)	168 (69)	123 (69)	69 (65)	154 (63)	108 (61)
III	30 (28)	75 (31)	55 (31)	37 (35)	92 (37)	68 (38)
IV	1 (1)	1 (0.4)	0 (0)	0 (0)	0 (0)	1 (0.6)

BMI, body mass index; LAP, laparoscopic surgery; OP, open surgery.

### 3.2 D3 dissection rates

Forty‐four patients were excluded from the evaluation of D3 dissection because of distant metastasis and severe invasion to adjacent organs, as were two patients with problems related to anesthetic management. Photographs were not submitted in 87 patients. Thus, the number of patients with analyzed photographs was 924, resulting in a submission rate of photographs for analysis of 91.4% (924/1011). The numbers of patients with analyzed photographs for each of the three periods for each approach were 462 for open surgery (1st period: 94, 2nd period: 212, 3rd period: 156) and 462 for laparoscopic surgery (1st period: 98, 2nd period: 210, 3rd period: 154). The photographs of five patients in the open surgery group and one patient in the laparoscopic group were unevaluable for analysis of D3 adequacy. Adequate D3 dissection was carried out in 95% or more of patients in both groups for each period. There were no changes in the rates of D3 dissection over time in either group (Table 2).

**Table 2:** D3 resection rates of patients in the present study

	1st period	2nd period	3rd period	*P*
OP (n = 462)				
Analyzed photographs (n)	94	212	156	
Adequate D3 (%)	94 (100)	210 (99.1)	149 (95.5)	0.823
Under D3 (%)	0 (0)	2 (0.9)	2 (1.3)	
Unevaluable (%)	0 (0)	0 (0)	5 (3.2)	
LAP (n = 462)				
Analyzed photographs (n)	98	210	154	
Adequate D3 (%)	95 (96.9)	209 (99.5)	149 (96.8)	0.117
Under D3 (%)	3 (3.1)	1 (0.5)	4 (2.6)	
Unevaluable (%)	0 (0)	0 (0)	1 (0.6)	

LAP, laparoscopic surgery; OP, open surgery.

*P* values were estimated by Fisher's exact test.

### 3.3 Short‐term outcomes

There were no significant changes over time in median operative time in either group (open surgery: 1st period: 160 minutes, 2nd period: 156 minutes, 3rd period: 161 minutes, *P* = 0.80; laparoscopic surgery: 1st period: 205 minutes, 2nd period: 211 minutes, 3rd period: 219 minutes, *P* = 0.37).

**Figure 2:**
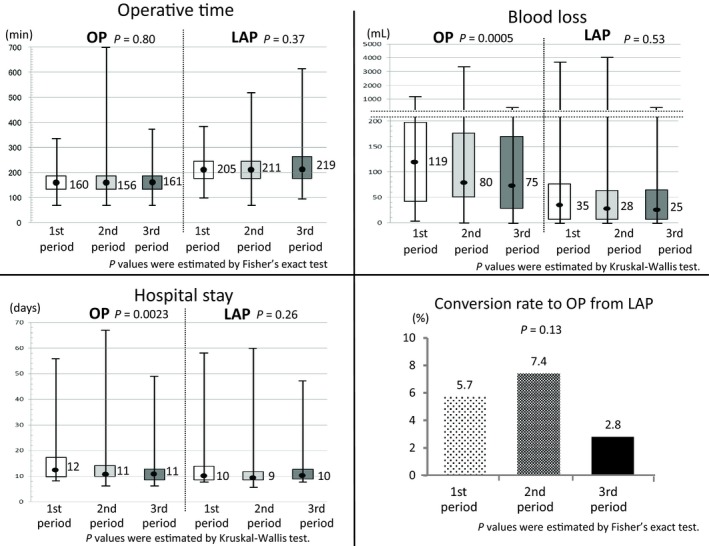
Change in short‐term outcomes and conversion rate to open from laparoscopic surgery over time. First period: October 2004 to December 2005, second period: January 2006 to December 2007, third period: January 2008 to March 2009. LAP, laparoscopic surgery; OP, open surgery

There was significant decrease of the median amount of blood loss in the latter periods in the open surgery group (1st period: 119 mL, 2nd period: 80 mL, 3rd period: 75 mL, *P* = 0.0005) statistically. However, it decreased gradually in the laparoscopic surgery group (1st period: 35 mL, 2nd period: 28 mL, 3rd period: 25 mL, *P* = 0.53). The change in the laparoscopic surgery group was not significant (Figure 2).

Length of hospital stay decreased in the latter periods in the open surgery group (1st period: 12 days, 2nd period: 11 days, 3rd period: 11 days, *P* = 0.0023) significantly, whereas it was consistently shorter in all periods in the laparoscopic surgery group (1st period: 10 days, 2nd period: 9 days, 3rd period: 10 days, *P* = 0.26) (Figure 2).

Conversion rate to open from laparoscopic surgery over time was non‐significant, although it was low in the 3rd period (1st period: 5.7%, 2nd period: 7.4%, 3rd period: 2.8%, *P* = 0.13) (Figure 2).

**Figure 3:**
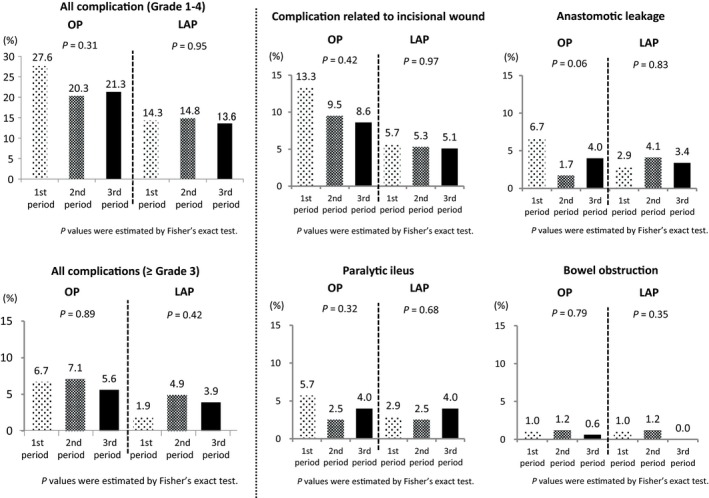
Change in early postoperative complication outcomes over time. First period: October 2004 to December 2005, second period: January 2006 to December 2007, third period: January 2008 to March 2009. LAP, laparoscopic surgery; OP, open surgery

Incidence of all grades of early complications decreased between the first period and the latter two periods in the open surgery group (1st period: 27.6%, 2nd period: 20.3%, 3rd period: 21.3%, *P* = 0.31). However, the incidence was consistently low in all periods in the laparoscopic surgery group (1st period: 14.3%, 2nd period: 14.8%, 3rd period: 13.6%, *P* = 0.95). There were no significant changes in the incidence of ≥grade 3 early complications over time in either group. In the analysis of the details of complications, there were no significant changes in any diseases in either group. Decreasing tendencies of incisional wound complications (1st period: 13.3%, 2nd period: 9.5%, 3rd period: 8.6%, *P* = 0.42) and anastomotic leakage (1st period: 6.7%, 2nd period: 1.7%, 3rd period: 4.0%, *P* = 0.06) were detected in the open surgery group, although they were not significant statistically. (Figure 3).

### 3.4 Long‐term outcomes

**Figure 4:**
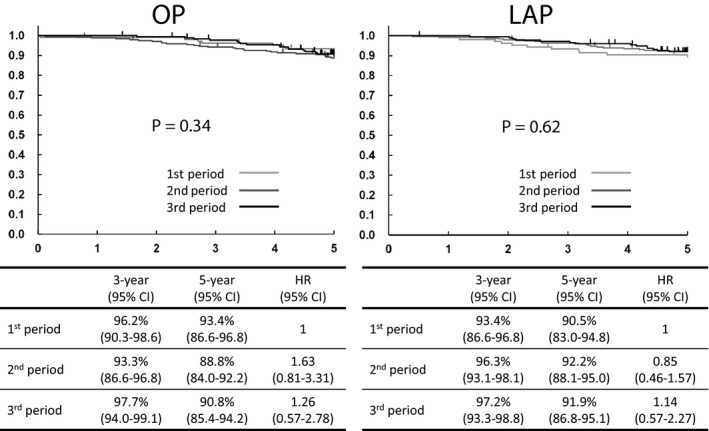
Overall survival. First period: October 2004 to December 2005, second period: January 2006 to December 2007, third period: January 2008 to March 2009. HR, hazard ratio; LAP, laparoscopic surgery; OP, open surgery

Estimated 5‐year rates of overall survival of open surgery were 93.4% (95% CI 86.6%‐96.8%) in the first period, 88.8% (95% CI 84.0%‐92.2%) in the second period, and 90.8% (95% CI 85.4%‐94.2%) in the third period. HR of open surgery were 1.63 (95% CI 0.81‐3.31) in the second period and 1.26 (95% CI 0.57‐2.78) in the third period. The estimated 5‐year rates of overall survival of laparoscopic surgery were 90.5% (95% CI 83.0%‐94.8%) in the first period, 92.2% (95% CI 88.1%‐95.0%) in the second period, and 91.9% (95% CI 86.8%‐95.1%) in the third period. HR of laparoscopic surgery were 0.85 (95% CI 0.46‐1.57) in the second period and 1.14 (95% CI 0.57‐2.27) in the third period. There was no particular trend in 5‐year overall survival based on the period (Figure 4).

**Figure 5:**
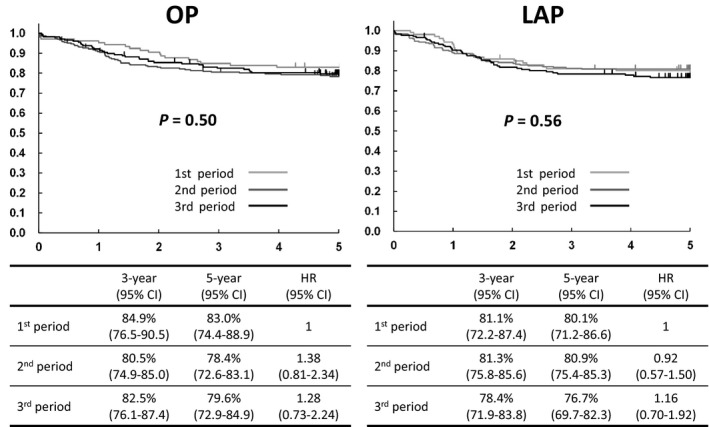
Relapse‐free survival. First period: October 2004 to December 2005, second period: January 2006 to December 2007, third period: January 2008 to March 2009. HR, hazard ratio; OP, open surgery; LAP, laparoscopic surgery

Estimated 5‐year rates of relapse‐free survival of open surgery were 83.0% (95% CI 74.4%‐88.9%) in the first period, 78.4% (95% CI 72.6%‐83.1%) in the second period, and 79.6% (95% CI 72.9%‐84.9%) in the third period. HR of open surgery were 1.38 (95% CI 0.81‐2.34) in the second period and 1.28 (95% CI 0.73‐2.24) in the third period. Estimated 5‐year rates of relapse‐free survival of laparoscopic surgery were 80.1% (95% CI 71.2%‐86.6%) in the first period, 80.9% (95% CI 75.4%‐85.3%) in the second period, and 76.7% (95% CI 69.7%‐82.3%) in the third period. HR of laparoscopic surgery were 0.92 (95% CI 0.57‐1.50) in the second period and 1.16 (95% CI 0.70‐1.92) in the third period. There was also no particular trend in 5‐year relapse‐free survival based on the period (Figure 5).

## 4 DISCUSSION

Nationwide permeation of laparoscopic colorectal surgery and development of chemotherapy were advanced during registration of the present study. The endoscopic surgical skill qualification system by JSES was started in 2004 and, on April 2005, the first endoscopically skilled surgeon was qualified by JSES. After 2008, the endoscopically skilled qualified surgeon was indispensable as a laparoscopic surgeon in charge. All participating hospitals finally had an endoscopically surgically skilled qualified surgeon. Some molecular targeted therapies for recurrence of colorectal cancer were covered by health insurance from the latter half of the registration period. For instance, bevacizumab was approved in June 2007, cetuximab in July 2008, and panitumumab in April 2010. Therefore, we expect that the change in short‐term and long‐term results was as a result of dividing the analysis into three periods rather than two periods.

Our data showed that among the short‐term outcomes, several parameters improved whereas others did not change in the latter periods in either group. Amount of blood loss and length of hospital stay significantly decreased in the latter periods in the open surgery group. The incidence of early complications also decreased in the latter periods in the open surgery group, but not significantly so. However, operative time did not change over time in either group. In the laparoscopic group, the amount of blood loss gradually decreased in the latter periods, whereas the length of hospital stay and the incidence of early complications did not change over time. Among the long‐term outcomes, there were no changes in the 5‐year rates of overall survival and relapse‐free survival in either group. To the best of our knowledge, this study is the first report to compare short‐term and long‐term results according to registration periods in a randomized study comparing laparoscopic and open surgery, and we also investigated maintenance of the quality of the surgical technique over time, especially in the laparoscopic surgery group.

A matched‐control study reported that operation time for laparoscopic surgery shortened in the latter period.^15^ Another study also reported shortened operation time in latter period, regardless of surgical experience or patient factors, as a result of the evolution of surgical apparatus.^16^ Therefore, we expected that the tendency for improvement would be especially remarkable in the short‐term outcomes of laparoscopic surgery. However, the results of the present study in laparoscopic surgery slightly contradicted our expectation. The continued development of surgical devices might not have had such a great influence on operation time.

Blood loss in the open surgery group was improved greatly in the latter periods, although it was invariably improved in the laparoscopic surgery group. As a reason for these results, it might be possible that the delicate and minute procedures carried out during laparoscopic surgery positively influenced surgical technique in open surgery. Advancements in laparoscopic technology allowed laparoscopic surgeons to carry out a refined procedure when considering the membrane structure of retroperitoneal anatomy.^17^ It is possible that knowledge of the minute and local anatomy gained from laparoscopic surgery regarding membrane structure was applied to open surgery. New energy devices such as ultrasonic coagulating shears and electrothermal bipolar vessel sealers also started to be used in laparoscopic surgery. These new energy devices reduce the amount of blood loss compared with standard electrical scalpels.^16^, ^18^ Ultrasonic scissors that are combined with a bipolar coagulation system have been shown to save operative time and enhance the safety of vessel ligation.^19^, ^20^ Significant change of the conversion rate to open from laparoscopic surgery was not detected over time. However, it was low in the third period. Therefore, there is a possibility that the development of energy device influenced conversion rate in the third period. We surmised that these devices were also used in open surgery, although we do not have the data to determine whether these new devices were actually used in open surgery. However, there were no contraindications to the use of new laparoscopic devices in open surgery.

The incidence of early complications decreased in the second period in the open surgery group and was almost equal between the second and third periods, although the change was not significant over time. In the open surgery group, the decreasing tendencies in the latter periods seemed to result from the decrease of incisional wound complications and anastomotic leakage. However, the reasons for this decrease are uncertain, and these were not significant differences.

Length of hospital stay decreased in the latter periods in the open surgery group. Many of the surgeons treated patients in both arms of this trial. It is common that postoperative management of laparoscopic surgery is programmed for a shorter length of hospital stay than that of open surgery. As the allocated procedure was not shielded from the investigators in this trial, investigators could notice early recovery from operation in both open surgery and laparoscopic surgery after experiencing postoperative management of laparoscopic surgery during the first and second periods. In fact, the length of hospital stay in the third period in the open surgery group was similar to that in the laparoscopic surgery group, whereas in the first and second periods, it was longer than that in the laparoscopic surgery group. Postoperative management in the laparoscopic surgery group might have favorably influenced the length of hospital stay in the open surgery group.

There was no particular trend in overall survival based on the periods in the two groups. Median survival time of patients with metastatic disease and/or recurrent colorectal cancer has gradually been extended by the development of chemotherapy in recent years.^21^, ^22^, ^23^ Therefore, we expected the overall survival of the patients in the latter two periods to be improved. In fact, the chemotherapy regimens used in this study may not have actually changed. Otherwise, changes of chemotherapy might not have influenced overall survival because there were few deaths in the trial. It was uncertain whether chemotherapy to treat recurrence was changed in this study because data on post‐recurrence therapy were not collected. A factor related to the invariable rates of relapse‐free survival over time might be the high rate of D3 dissection. Submission of a photograph of the operative field was required in this trial, and this might have caused an increase in the D3 dissection rate that potentially resulted in the excellent long‐term prognosis.^24^

One important factor influencing both the short‐ and long‐term outcomes was associated with the learning curve for surgical treatment. In the present trial, participation in at least 30 laparoscopic surgeries was required. Although it might be debatable as to whether 30 surgeries is an appropriate number, research into the learning curve in the first half of the 2000s, and also in recent reports, found that laparoscopic procedures stabilized when surgeons had experienced approximately 30 procedures.^25^, ^26^ Certification by the endoscopic surgical skill qualification system introduced by JSES has been required since 2 years after the first patient was registered in this trial (2006).^27^ Qualified surgeons were shown to improve the safety of laparoscopic surgery in urology and pediatric surgery.^28^, ^29^ Especially for colorectal surgery, the safety of laparoscopic surgery might be adequately guaranteed under the supervision of a surgeon qualified by JSES.^30^ The endoscopic surgical skill qualification system of JSES originated in Japan, and certification is very difficult to obtain. Therefore, we surmised that the quality of laparoscopic surgery was maintained throughout the trial.

In the CLASICC study, the conversion rate improved from 38% in the first year of registration to 16% in the final year.^3^ Difference in short‐term outcomes according to registration period was reported in a case‐matched study.^15^ There was some anxiety over how the different registration periods would influence the results because laparoscopic surgery was a new procedure at the beginning of that study. However, there were no differences in short‐term and long‐term results between the periods in the laparoscopic surgery. The conversion rate of the present study was low at 5.4%, whereas it was reported to range between 10% and 20% in other randomized controlled studies.^1^, ^2^, ^3^, ^4^, ^5^ As the reason, it seems that severe regulation of laparoscopic surgeons influenced the results. In particular, the introduction of JSES‐qualified surgeons might have had a very large influence. A qualification system to evaluate technical skill appears to be important in clinical trials relating to surgical procedures.

There are some limitations in the present study. First, we do not have any data on the energy devices used in both arms of the trial. Therefore, the influence of technological improvements on the outcomes can only be surmised. Second, we do not have data on chemotherapy given for recurrences in each period. In fact, we believe that neither the development of surgical devices nor the improvement of chemotherapy influenced the short‐ and long‐term results during the study period.

In conclusion, operation time between the open surgery group and the laparoscopic surgery group in the JCOG0404 trial did not change over time, although blood loss in the open surgery group improved in the latter two periods. The long‐term results did not change over time in either group. The quality control applied in this trial might have been effective in producing such safe endpoints.

## DISCLOSURE

Conflicts of Interest: Authors declare no conflicts of interest for this study.
